# Digitally embodied lifespan neurocognitive development and Tactile Internet: Transdisciplinary challenges and opportunities

**DOI:** 10.3389/fnhum.2023.1116501

**Published:** 2023-02-10

**Authors:** Shu-Chen Li, Frank H. P. Fitzek

**Affiliations:** ^1^Chair of Lifespan Developmental Neuroscience, Faculty of Psychology, Technische Universität Dresden, Dresden, Germany; ^2^Centre for Tactile Internet With Human-in-the-Loop, Technische Universität Dresden, Dresden, Germany; ^3^Deutsche Telekom Chair of Communication Networks, Faculty of Electrical and Computer Engineering, Technische Universität Dresden, Dresden, Germany

**Keywords:** development, neuromodulation, signal-to-noise, perception, multisensory, sensory augmentation, Tactile Internet, aging

## Abstract

Mechanisms underlying perceptual processing and inference undergo substantial changes across the lifespan. If utilized properly, technologies could support and buffer the relatively more limited neurocognitive functions in the still developing or aging brains. Over the past decade, a new type of digital communication infrastructure, known as the “Tactile Internet (TI),” is emerging in the fields of telecommunication, sensor and actuator technologies and machine learning. A key aim of the TI is to enable humans to experience and interact with remote and virtual environments through digitalized multimodal sensory signals that also include the haptic (tactile and kinesthetic) sense. Besides their applied focus, such technologies may offer new opportunities for the research tapping into mechanisms of digitally embodied perception and cognition as well as how they may differ across age cohorts. However, there are challenges in translating empirical findings and theories about neurocognitive mechanisms of perception and lifespan development into the day-to-day practices of engineering research and technological development. On the one hand, the capacity and efficiency of digital communication are affected by signal transmission noise according to Shannon’s (1949) Information Theory. On the other hand, neurotransmitters, which have been postulated as means that regulate the signal-to-noise ratio of neural information processing (e.g., Servan-Schreiber et al., 1990), decline substantially during aging. Thus, here we highlight neuronal gain control of perceptual processing and perceptual inference to illustrate potential interfaces for developing age-adjusted technologies to enable plausible multisensory digital embodiments for perceptual and cognitive interactions in remote or virtual environments.

## 1. Introduction

Human perceptual and cognitive processes are embodied through the sensory systems and embedded in physical, technological, and socio-cultural contexts (Quartz, 1999; Clark, 2012). Through perceiving different types of sensory information (e.g., auditory, visual, and tactile) and behaving in various environments, humans learn through their experiences to adapt to contextual constraints and affordances. At the same time, humans also modify the environments to construct new conditions for their lives, to which eventually require new adaptations. In this manner, human development at the individual and societal levels necessitates co-constructive processes between adaptive neurobiological and psychological mechanisms, on the one hand, and collectively generated resources for supporting individual development and general human conditions, on the other hand (Li, 2003).

A powerful collective human capacity in such “niche construction” processes (cf., Laland et al., 1999) is technological advancements that bring forth new tools, instruments, and infrastructures to alter life conditions. Since the rise of modern computers in the 1940s (Ifrah, 2000), the continued developments in computer, telecommunication, and other digital technologies have created digital environments for human behaviors and the research about them. Over the past decades the Internet and World Wide Web (Wolinsky, 1999) have become key digital infrastructures for remote access and exchange of information *via* the visual and auditory modalities. Furthermore, technologies of virtual and augmented reality (VR and AR) provide a spectrum of digital contexts that not only have the potentials for medical, educational, and industrial applications (see Eden et al., 2022 for review), but also as research tools for lab-based experiments to allow well-controlled naturalistic studies of human behaviors (e.g., Brookes et al., 2020; Hofmann et al., 2021). However, these technologies currently still constrain the digitally embodied perception and cognition mainly to the visual and auditory modalities. To go beyond such limitations, the means of digital information processing and communication need to expand to also include other modalities.

### 1.1. Toward new technologies for digitally transmitted perception and cognition

**FIGURE 1:**
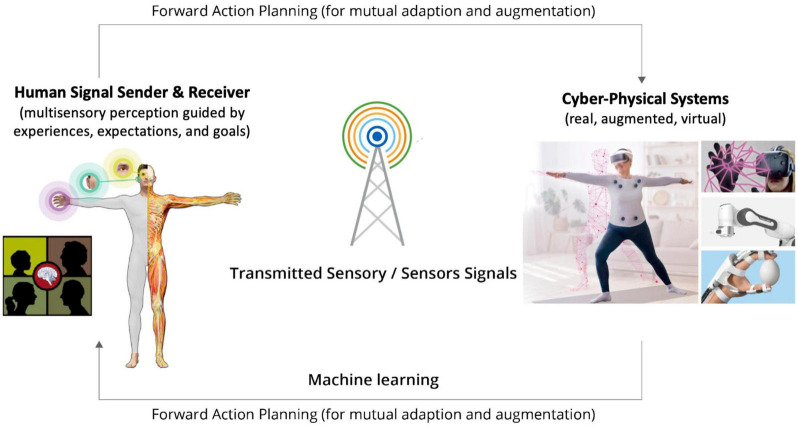
Schematic diagram of digitally transmitted closed-loop perceptual and cognitive interactions between humans and cyber physical systems (CPS) supported by Tactile Internet (TI) technologies [parts of the image are adapted from the authors’ work, Li et al., 2021, Copyright Elsevier 2021; the image of the senior female is a modification of royalty-free content with usage licence, credit: Prostock-studio/shutterstock.com].

Recent progresses in digital communication, sensor, and actuator technologies, as well as machine-learning algorithms have stipulated the emergence of a new type of digital communication infrastructure, known as the Tactile Internet (TI), that goes beyond audio-visual based information exchanges (see Simsek et al., 2019; Fitzek et al., 2021 for reviews). Specifically, the TI is defined as: “A network or network of networks for remotely accessing, perceiving, manipulating or controlling real or virtual objects or processes in perceived real time by humans or machines.” The TI technologies aim to enable digitally transmitted closed-loop human-human or human-machine interactions in quasi real time by providing new multisensory interaction avenues for broad populations of users to experience and control remote, VR/AR, or other combinations of digitalized environments (Figure 1). Such interaction interfaces include, but not limited to, digitalized haptic (tactile and kinesthetic) information and cyber physical systems (CPS, i.e., systems of software and devices).

When TI technologies are advanced enough to provide plausible digital sensory augmentations and stable remote interactions in quasi-real time, they could be applied to support human behavior in daily lives, particularly for populations whose perceptual and cognitive processes are either still undergoing development or affected by aging-related declines. Immersive VR, AR, or mixed-reality technologies may also open new solutions for designing more naturalistic experimental studies for cognitive neuroscience research. However, a key challenge in developing such technologies lies in concretely realizing the “human-in-the-loop” approach (Schirner et al., 2013) to integrate principles of human perception and cognition into engineering research and technological development. To this end, the research on lifespan neurocognitive development needs to take up transdisciplinary challenges of collaborating with the engineering fields of sensor and actuator as well as telecommunication technologies, beyond usual collaborations with cognitive scientists, neurobiologists, and medical researchers.

### 1.2. Digitally embodied closed-loop perceptual and cognitive interactions

Through TI technologies and the more recent vision of digitalized immersive 3D realms known as Metaverse (e.g., Al-Ghaili et al., 2022; Mystakidis, 2022; Rostami and Maier, 2022), the embodiments of human perceptual and cognitive processes would frequently be interacting with digitalized multisensory signals including haptics. Such “digital embodiments” raise challenges for digitalizing sensory signals in ways that preserve key stimulus characteristics for humans to sense and perceive, while not overburdening the latency and capacity requirements on the telecommunication networks that transmit these signals (e.g., Steinbach et al., 2018; Noll et al., 2022). The requirement of low latency is particularly important for remote closed-loop interactions to operate in a quasi-real-time manner and remain stable (e.g., Bachhuber and Steinbach, 2015). Another challenge is that the digitalized signals may not be perceived equally well and plausible either by the same person in different situational contexts or by people of different ages. Thus, it is important to integrate psychophysical and neurocognitive mechanisms of multisensory perception (e.g., Ernst and Banks, 2002; see Ernst and Bülthoff, 2004; Macaluso and Driver, 2005 for reviews) as well as their development and aging (Burr and Gori, 2012; Li and Rieckmann, 2014; Stein et al., 2014; Klever et al., 2019; Nava et al., 2020; Jones and Noppeney, 2021) into the very processes of designing sensor, augmentation, and network technologies for digitally transmitted perceptual and cognitive interactions.

## 2. Noise in digital communication and neuronal gain control of human information processing

**FIGURE 2:**
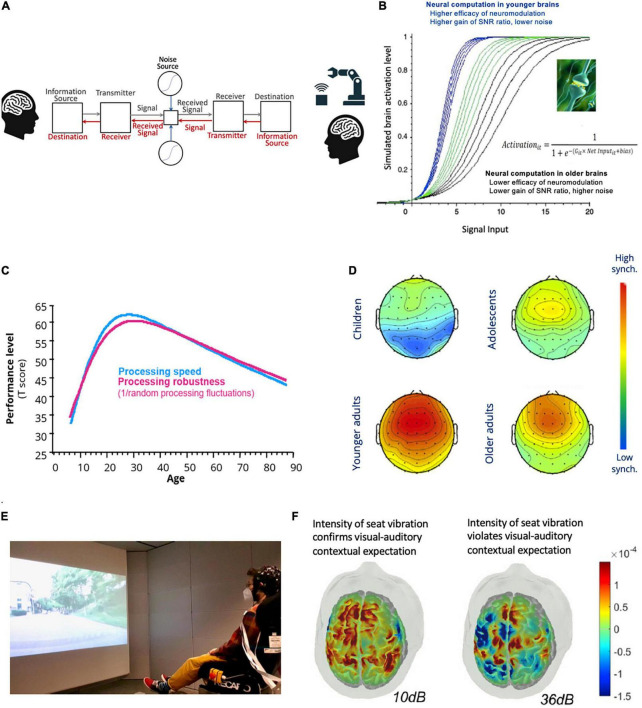
Interfaces for transdisciplinary research. **(A)** Digitally embodied closed-loop human-machine or human-human communication system in the presence of physical and biological noise. **(B)** Varying values of the G parameter of artificial neurons simulates dopaminergic neuronal gain control of neural information processing in younger and older adults (see text and Li et al., 2001, 2006 for details). **(C)** The speed and robustness of cognitive and perceptual processing vary across the lifespan [see text for details; figure plotted with data from the author’s work Li et al. (2004)]. **(D)** Inter-trial phase coherence of EEG frontal theta activities differs across the lifespan [see text for details. Images adapted from Papenberg et al. (2013), with permission, Copyright Elsevier 2013]. **(E)** Assessing cortical processes of contextual expectancy modulation of tactile perception using fNIRS in a virtual driving environment with audio-visual scenes of different road types (shown here is a scenario of driving on smooth road). **(F)** Plausible vibrotactile stimulations at a lower intensity but confirm with contextual expectation based on audio-visual information in the virtual driving scene elicited greater cortical responses in the sensorimotor cortex than implausible stimulations at a higher intensity [see text for details; images in **(E)** and **(F)** are adapted with permission from Kang et al. (2022), Communications Biology, 5:1360, 1–13].

Neither humans nor the CPS can be expected to “perceive and act” with perfect precision under all conditions. Sensory or sensor noise as well as process unreliability and delays are inevitable, be it from the biological or technological systems (Figure 2A). The potential amplifications of multiple sources of noise, unreliability, and delays are big challenges for realizing real-time digitally transmitted remote human-human or human-machine interactions that also entail haptic signals.

The fact that noise attenuates signal resolution and the capacity of digital communication channels (e.g., Diggavi and Cover, 2001; van Wyk et al., 2021) is well known since Shannon’s (1949) classical information theory. During digitally transmitted closed-loop interactions, other than channel noise arising from telecommunication infrastructures, fluctuations in neural information processing are an intrinsic noise source in the brains (Manwani and Koch, 1999; Deco et al., 2009; Ferguson and Cardin, 2020) of the human information senders and receivers. It is, therefore, important to consider mechanisms that regulate neural information processing fidelity and how they may change during brain development and aging, since the new TI and related technologies envision to support broad populations of users.

Sensory signals usually can be noisy and the perceptual environments entail certain degrees of uncertainty (Ernst and Bülthoff, 2004). The human brains carry out complex computations to process sensory signals from the environment for us to consciously perceive and form internal models about important aspects of the surrounding world, which then allow us to make perceptual inferences for guiding behavior (e.g., Friston et al., 2012; Hoffman, 2019). Besides propagating activation potentials (i.e., nerve impulses in the form of tiny electric charges in the range of millivolts) through the axons in neurons, neural information processing also requires chemical signal transmissions between neurons. A variety of chemical substances, known as neurotransmitters, carry signals for information exchanges between neurons (see Hyman, 2005 for review). It is estimated that the human brain has about 10^11^ neurons, with each having 1,000–10,000 synapses, also known as connections (Azevedo et al., 2009; Zimmer, 2011). Electrical and neurochemical communications between neurons occur at the around 10^14^–10^15^ synapses the human adult brain has (Pereda, 2014).

Among the various neurotransmitters, dopamine is the focus here for several reasons. First, other than striatal dopamine’s well-known role in signaling reward values (Schultz, 2016), it also codes sensory and motor information (Engelhard et al., 2019) and regulate top-down cognitive control functions (Ott and Nieder, 2019). Together these make the dopamine system important for cognitive and perceptual functions (Lerner et al., 2021). Second, computational theories of neuronal gain control suggest that dopamine plays important roles in regulating the signal-to-noise ratio (SNR) of neural information processing (Figure 2B; Li et al., 2001; Servan-Schreiber et al., 1990) and in regulating the balance between bottom-up sensory saliency and top-down prior expectations during perceptual processing and inference (Friston et al., 2012; Pezzulo et al., 2018). Third, empirical evidence shows that dopamine affects performance fluctuations and the precision of brain activities (e.g., MacDonald et al., 2012; Yousif et al., 2016). Fourth, dopamine modulation is important for tactile perception (e.g., de Lafuente and Romo, 2011) and changes across the lifespan (see Li and Rieckmann, 2014 for review).

### 2.1. Lifespan development of dopaminergic gain control of processing fidelity

Empirical data based on receptor imaging of children or adolescents (e.g., Jucaite et al., 2005) are very scarce, due to the use of radioactive ligands in such imaging technique. However, non-invasive techniques of assessing protein expressions of dopamine receptors have shown very gradual development of dopamine receptors. After birth, protein expressions of the dopamine D1 receptor, important for top-down cognitive control and working memory functions in the prefrontal cortex (Takahashi et al., 2012), undergo gradual increase during childhood and adolescence and only reach the highest level in young adulthood (Rothmond et al., 2012). As for aging-related decline, accumulated receptor imaging findings indicate that although dopamine synthesis capacity is not affected much by aging (Karrer et al., 2017), the densities of dopamine D1 and D2 receptors as well as the presynaptic dopamine transporter (DAT) decline substantially during adulthood, with estimates of about 10% receptor losses per decade, starting from mid 20s (Erixon-Lindroth et al., 2005; Inoue et al., 2001; Kaasinen and Rinne, 2002).

Other than modeling dopamine as the reward prediction signal as in reinforcement learning algorithms (Dayan and Niv, 2008; Glimcher, 2011), dopaminergic modulation has also been modeled as neuronal gain control of the SNR of information transmission between neurons. Specifically, the gain (G) parameter of the sigmoidal (also termed logistic) input-output function (Figure 2B) can be adjusted to simulate individual (e.g., Servan-Schreiber et al., 1990) or age-related differences in dopamine levels (e.g., Li et al., 2001, 2006). Lowering the values of the G parameter to simulate lower levels of dopamine receptors in aging brains (Kaasinen et al., 2000; Erixon-Lindroth et al., 2005) reduces the slopes and non-linearity of the sigmoidal function, which consequently reduces the SNR of activation propagations across layers of simulated artificial neurons and limits their computational complexity (Li and Sikström, 2002). Functionally, attenuating gain control in neural network models results in lower processing fidelity that is reflected in larger activation fluctuations when responding to identical inputs as well as less distinctive internal activation patterns stored in the network’s connections that are triggered by different external input stimuli (Li and Sikström, 2002). Moreover, a net effect of varying the slope and the continuous non-linearity of the sigmoidal function by either reducing or excessively increasing the G parameter also constraints the network’s memory storage capacity (see Li and Rieckmann, 2014 for review), which is in line with the empirically observed inverted-U function of dopamine modulation of frontal cognitive processes (Cools and D’Esposito, 2011). Given these computational effects, it can be surmised that the gradual development of dopamine D1 receptor protein and aging-related declines of dopamine receptors suggest, respectively, less mature or declined neuronal gain control of information transmissions in developing and aging brains. These would have functional consequences on behavioral and brain processing fidelity.

Indeed, not only that cognitive and perceptual processing speed is slower in children and older adults, their processing speeds also fluctuate more across time (Figure 2C; Li et al., 2004; Papenberg et al., 2013). This reflects less reliable or less robust information processing in terms of brain dynamics measured with electroencephalography (EEG). For instance, the inter-trial phase coherence reflecting synchronicity of neuronal activities across time is lower in children, adolescents, and older adults than in younger adults (Figure 2D). Furthermore, within each of age groups lower levels of EEG synchronicity are associated with less reliable processing speed, indicating that higher behavioral fluctuations reflect lower temporal precision of brain electrophysiological activities (Papenberg et al., 2013).

### 2.2. Dopaminergic modulation of tactile perception

The impacts of dopamine and other related monoaminergic transmitters (serotonin and noradrenaline) on the coding and subsequent perception of sensory signals have been investigated with respect to different modalities in animals (Bao et al., 2004; Engelhard et al., 2019; see Jacob and Nienborg, 2018 for review) and in humans (e.g., Li et al., 2013; Yousif et al., 2016; Beste et al., 2018; Bang et al., 2020). We focus here on its role in modulating tactile perception.

Evidence from animal research shows that when detecting vibrotactile stimulations, the firing rates of neurons in several brain regions of Rhesus monkeys vary with the intensities and temporal characteristics of the vibrotactile signals (Lebedev et al., 1994; de Lafuente and Romo, 2006). Moreover, while anticipating the stimuli, the firing rate of midbrain dopamine neurons codes the uncertainty about the presence or absence of the tactile signals (de Lafuente and Romo, 2005) and reflects stimulation amplitude when the presence of a tractile stimulus is detected, indicating that dopamine modulates subjective tactile experiences (de Lafuente and Romo, 2011). The response time course of midbrain dopamine neurons during vibrotactile perception matches more closely to the onset of perceptual processes in the frontal premotor cortex (de Lafuente and Romo, 2005), instead of neuronal activities in the somatosensory cortex (de Lafuente and Romo, 2011). Together, these findings indicate that dopamine neurons underlie (i) the subjective experience of the perceived, rather than mere sensory, aspects of tactile signals and (ii) the regulation of inherent uncertainty during perceptual processing and inference. Furthermore, evidence from a recent receptor imaging study (Schneider et al., 2019) with a larger sample of monkeys also shows that the binding potentials of dopamine receptors (D1 and DAT) correlate with behavioral responsivity to tactile stimuli. Human pharmacological research also found that increasing the level of monoamines in the brain by administering amphetamine increases perceptual and cortical plasticity of tactile learning (Dinse et al., 2003).

## 3. Contextual expectancy and plausible sensory augmentation

Besides effects of neuronal gain control on sensory coding precision, the perceptual system needs to infer the most likely percept the sensory signals may represent. A principle of perception since Helmholtz’s (1857) classical view is that perception is guided by expectations that are based on prior experiences in similar contexts. In modern cognitive science, it is also recognized that external sensory signals alone are not sufficient to represent reality, instead individuals are conscious agents whose experiences may guide perceptual processing of sensory inputs to construct and reconstruct the perceived reality (Hoffman, 2019). Multisensory perception could be driven by low-level stimulus properties (e.g., temporal contingency, spatial congruence) or high-level contextual information (e.g., semantic relatedness, situational schemas, or multisensory scene context). Furthermore, the bottom-up sensory signals and top-down expectations interact during perception (Ernst and Bülthoff, 2004; Chen and Spence, 2010; Deroy et al., 2016; Gau and Noppeney, 2016; de Lange et al., 2018). Bayesian inference theories of perception and action posit that, through regulating the SNR of information processing, dopamine also influences the balance between a person’s prior expectations in a certain situational context and the saliency of sensory inputs from the environment (Friston et al., 2012; Pezzulo et al., 2018). Pathologies involving malfunctions of the dopamine system could result in a faulty bias of overweighting prior expectations, which lead to hallucinatory percepts such as in the cases of schizophrenia (Cassidy et al., 2018) or Parkinson’s disease (Collerton et al., 2012).

### 3.1. Contextual expectations modulate multisensory perception

Impacts of contextual expectancy on multisensory perception have been investigated in several modalities. For instance, when processing audio-visual stimuli, hearing semantically related auditory information (e.g., a barking sound) triggers the expectation of a certain animal (e.g., image of a dog) and enhances the identification of unclear or degraded visual images (Chen and Spence, 2010). As for visual-taste perception, seeing colors that confirm with the expectations of flavors of certain fruits enhances the accuracy of flavor perception (Spence et al., 2010). Furthermore, past studies also indicated that expectancy could induce changes in perceptual representations beyond just biasing the threshold of perceptual decisions. Cortical responses in the primary taste cortex after drinking mildly sweet drinks are stronger when individuals are led to expect a very sweet drink relative to without such an expectation (Woods et al., 2011). In terms of pain perception, which could be shaped by multisensory signals (Senkowski et al., 2014), a placebo drug for presumed pain relief was found to yield lower responses in brain regions of pain processing (e.g., thalamus and insula) and enhance activities in prefrontal regions associated with the anticipation of pain. Negative correlations between placebo-induced increase of cortical activities in the frontal expectancy regions and placebo-induced reduction of activities in regions of pain processing suggest that contextual expectations modulate pain perception (Wager et al., 2004).

### 3.2. Toward neurocognitive mechanisms of plausible tactile augmentation

Thus far, evidence for expectancy modulation of tactile perception is much scarcer than for other modalities. However, a recent study manipulated contextual congruency between audio-visual information of driving scenes and the intensity of vibrotactile augmentation in a VR environment to investigate cortical processes underlying plausible sensory augmentation in young adults (Kang et al., 2022). Videos of driving scenes with smooth (e.g., highway) or rough (e.g., cobblestone) road surfaces along with the corresponding audio sounds were paired either with strong or low vibrotactile stimulations delivered from a car seat (Figure 2E). Cortical activities in the frontal and sensorimotor regions that were measured with functional near-infrared-spectroscopy (fNIRS) were found to be stronger when responding to plausible vibrotactile stimulations with intensities at levels that could be expected, given the audio-visual information in the virtual scenarios. In line with findings for other sensory modalities, contextual expectancy in this case also affected tactile perceptual representations. Vibrotactile stimulations of a lower intensity but confirms with contextual expectations based on audio-visual information resulted in greater cortical activities in the sensorimotor cortex than stronger but implausible stimulations (Figure 2F). Furthermore, frontal activities under expected scenarios correlate negatively with expectation violation costs in the sensorimotor cortex, which indicate frontal top-down expectancy regulation of tactile perceptual representation.

The extent to which virtual events conform to human expectations – i.e., the concept of plausibility – is a commonly used criterion for constructing virtual environments that could be perceived as sufficiently realistic (Slater, 2009). The notion of defining the “plausibility” of virtual events in relation to the person’s expectations in VR research is very much in line with Bayesian theories and empirically observed effects of expectancy modulation of perception. Further transdisciplinary collaborations between neurocognitive research on expectancy modulation of perception and sensing/actuating technologies for VR/AR applications would be helpful to understand mechanisms underlying (multi)sensory augmentations for designing plausible digital embodiments. Besides machine-learning algorithms and network communication infrastructures, these technologies are also crucial components for the envisioned Metaverse (e.g., see Al-Ghaili et al., 2022 for review), which aims at developing digitalized immersive 3D realms that can flexibly combine real and different types of virtual environments (AR and mixed-reality) for multi-agent (human-human and human-machine) exchanges. In this regard, perceptual and lifespan developmental neuroscience research could guide the designs of AR/VR devices and other mixtures of digitalized environments to suit the neurocognitive functions of broad populations of users. Below, we highlight two lines of collaborative interfaces.

For instance, it has been demonstrated that empirical data of human psychophysical studies of perceptual judgments help the selection of different tactile codecs (e.g., Muschter et al., 2021), i.e., protocols for compressing digitalized tactile signals, for standardization. However, as of yet, it is not clear whether compressed tactile signals can be perceived equally well by people of different ages. A recent pilot study with a small sample of younger and older adults showed lager between-person differences in perceptual judgments of compressed vibrotactile signals in older than in younger adults, indicating that a given tactile codec might not yield satisfactory performance for all users in the older populations (Muschter et al., 2022). Given dopamine’s role of neuronal gain control affecting the SNR of neural information processing (Servan-Schreiber et al., 1990; Li et al., 2001, 2006), its function in coding uncertainty and amplitude of tactile stimulations (de Lafuente and Romo, 2006, 2011), as well as the clear aging-related declines of dopamine receptors (Kaasinen et al., 2000; Erixon-Lindroth et al., 2005), a potential interface is to conduct systematic age-comparative studies of perceptual judgments of compressed sensory information by different haptic codecs (Steinbach et al., 2018) to provide empirical data for age-adjusted codec designs and standardization.

Another potential interface is collaborative research on the design of plausible multisensory experiences in AR/VR or other mixtures of digital environments as envisioned in Metaverse (e.g., Al-Ghaili et al., 2022). In this regard, neurocognitive mechanisms of contextual expectancy-based multisensory perception presented above (Kang et al., 2022) is a starting point for further studies on foundations for plausible subjective experiences in digitalized environments. The frontal cortex underlying top-down expectancy-based control decline substantially during aging at the anatomical (e.g., Raz et al., 2005), functional (e.g., Nyberg et al., 2010), and neurochemical (e.g., Kaasinen et al., 2000; see Li and Rieckmann, 2014) levels. Moreover, the trade-off of dopamine modulation in the frontal and striatal regions underlie effects of uncertainty and prior expectations on cognitive control of flexible behavior (Daw et al., 2005) as well as perceptual inference (Friston et al., 2012; Cassidy et al., 2018). Thus, systematic collaborative age-comparative research on neurocognitive correlates of plausible perceptual experiences in AR/VR would be helpful for further developing other 3D immersive digital environments.

## 4. Concluding remark: Toward age-adjusted digital embodiment technologies

Current advancements in several subfields of TI technologies aim to develop new digital infrastructures to also integrate haptic signals for humans to remotely interact with other humans or machines, beyond just being able to hear or see the interaction partners. These technologies bare the potentials in generating new ways of supporting different aspects of human life, including applications in educational settings and gerontological care. However, current issues regarding plausible renderings of digitalized multisensory signals as well as network requirements in the speed, capacity, and reliability of transmitting large amounts of multisensory data are challenges that need to be resolved. Furthermore, age-related differences in perceptual and cognitive functions are usually not systematically evaluated en route to technological developments, which may limit the scope of potential user populations. As presented here, since the efficacy of dopaminergic modulation matures rather gradually during development and decline substantially during aging, the precision of registering and making perceptual inference of digitalized sensory signals would also be lower in these populations. It is thus important to systematically scrutinize age-related differences in neuromodulation of perceptual processing and perceptual inference, particularly in multisensory tasks involving the haptic sense, to inform age-adjusted development of TI technologies that can also support children and seniors in digitally embodied perceptual and cognitive interactions.

Other than the two potential interfaces selectively highlighted in this paper, collaborative research on lifespan differences in mechanisms underlying the perception of social affective touch (e.g., Croy et al., 2019; McIntyre et al., 2021) is another interface for developing actuation technologies of touch (e.g., Muthukumarana et al., 2020) that are important for digitally transmitted social interactions. In addition, besides the plausibility of digitalized sensory signals, the subjective feeling of personal presence is another ingredient for creating immersive digital environments. In this regard, cognitive neuroscience research that compare peri-personal space and body illusion in real and virtual environments offer another potential interface (see Serino, 2019 for review). Lastly, cognitive and neuroscience research on representations of joint action (see Shea et al., 2014; Prinz, 2015; for review) and the associated age differences (e.g., Keitel et al., 2014) as well as the research on making active inference in social situations (e.g., Friston and Frith, 2015; Hipólito and van Es, 2022) is another interface for designing algorithms for digitally transmitted multi-person/agent interactions.

## Data availability statement

The original contributions presented in this perspective article are included in the references, further information can be directed to the corresponding author.

## Author contributions

S-CL and FF collaborated extensively in several transdisciplinary projects, edited the manuscript together and approved the submitted version. S-CL drafted the manuscript based on their collaborations. Both authors contributed to the article and approved the submitted version.
